# Patient-derived tumoroids and proteomic signatures: tools for early drug discovery

**DOI:** 10.3389/fimmu.2024.1379613

**Published:** 2024-04-18

**Authors:** Hélène Lê, Jules Deforges, Pasquale Cutolo, Anissa Lamarque, Guoqiang Hua, Véronique Lindner, Shreyansh Jain, Jean-Marc Balloul, Nadia Benkirane-Jessel, Eric Quéméneur

**Affiliations:** ^1^ Transgene S.A., Illkirch–Graffenstaden, France; ^2^ INSERM UMR1260, Regenerative Nanomedicine, Strasbourg, France; ^3^ Olink, Uppsala, Sweden; ^4^ Department of Pathology, Hopitaux Universitaires de Strasbourg, Strasbourg, France

**Keywords:** onco-virotherapy, Anti-cancer Therapy, immuno-oncology, non-small-cell lung cancer, patient-derived tumoroids, proteomic

## Abstract

Onco-virotherapy is an emergent treatment for cancer based on viral vectors. The therapeutic activity is based on two different mechanisms including tumor-specific oncolysis and immunostimulatory properties. In this study, we evaluated onco-virotherapy *in vitro* responses on immunocompetent non-small cell lung cancer (NSCLC) patient-derived tumoroids (PDTs) and healthy organoids. PDTs are accurate tools to predict patient’s clinical responses at the *in vitro* stage. We showed that onco-virotherapy could exert specific antitumoral effects by producing a higher number of viral particles in PDTs than in healthy organoids. In the present work, we used multiplex protein screening, based on proximity extension assay to highlight different response profiles. Our results pointed to the increase of proteins implied in T cell activation, such as IFN-γ following onco-virotherapy treatment. Based on our observation, oncolytic viruses-based therapy responders are dependent on several factors: a high PD-L1 expression, which is a biomarker of greater immune response under immunotherapies, and the number of viral particles present in tumor tissue, which is dependent to the metabolic state of tumoral cells. Herein, we highlight the use of PDTs as an alternative *in vitro* model to assess patient-specific responses to onco-virotherapy at the early stage of the preclinical phases.

## 1 Introduction

Preclinical research has developed an outgrowing interest in the development of human-based models. Indeed, there is a consciousness that current animal models don’t accurately recapitulate human features such as anatomy, physiological barriers, receptor panels, physiopathology mechanisms, and especially, immune responses (1, 2). Conventional oncology models mainly comprise murine models and present a low predictive value of human responses (1). For example, in patient-derived xenografts (PDXs) models, the main limitations are (i) human cells that are progressively replaced by stromal murine cells, (ii) the lack of immune system, and (iii) the lack of tumor cell interactions with human-relevant stroma, that represent a functional tumoral microenvironment (TME) (3). Even though murine models contain a functional immune system, there’s a poor clinical prediction of human immune responses. These current oncology models fail to detect the risk of drug inefficiency and safety issues. Nearly half of the drug candidates fail in clinical phases due to the lack of efficacy (4). Regarding the lack of safety issues detection in preclinical studies, they represent 30% of candidate drug failure (4). These clinical issues lead to a high attrition rate, reaching 95% in 2021 for all therapeutic areas (5, 6). In oncology, the attrition rate is 2 to 4 times more important compared to other therapeutic areas, as reported from 1979 to 2014 (7).

To better predict therapeutic effects at the early stages of drug discovery, researchers are putting efforts on human-based models to propose a predictive and relevant preclinical model that could complement current animal models (8). Different strategies have been developed to bridge these inter-species differences and increase the link between preclinical and clinical phases. In the last decade, organoids and organ-on-chips have been widely described (9). Furthermore, the latest amendment of the Food and Drug Administration (FDA) supports the use of alternative *in vitro* models when animal models are not required, matching with the 3Rs approach (10). Organoids are described as self-organizing 3D structures that mimic a specific organ. To better reflect the primary tumoral tissue derived from patients, patient-derived tumoroids (PDTs) were developed. For example, in lung cancer, numerous models of PDTs have been developed. Yet, some limitations, such as the lack of stromal and immune cells in the TME, can be noted (11).

We have previously developed a vascularized immunocompetent model of patient-derived tumoral organoids that modeled the immune part of the TME by introducing immune cells derived from peripheral blood. Co-culture with peripheral blood mononuclear cells (PBMCs) was used to assess T cell infiltration within PDTs after a treatment (12). However, PBMCs are less predictive compared to tumor-infiltrated immune cells. As tumor-infiltrated immune cells are in contact with tumoral cells, a predictive value of these cells has been assigned to immunotherapy responses (13). For example, for non-responders to immunotherapy, an increased proportion of myeloid-derived suppressor cells (MDSCs), and a decreased proportion of natural killer (NK) cells, and monocytes in the tumor site constitute an immunosuppressive microenvironment. As for responders to immunotherapy, the presence of high T-cell immunoglobulin and mucin-domain containing 3 (TIM-3), lymphocyte-antigen gene 3 (LAG-3), and programmed death-1 (PD-1) on immune cells within the stroma were reported in patients with better survival (14). Furthermore, they recapitulate T cell activation and tumor-killing to immunotherapy treatments (15, 16). We have evolved toward a model of PDTs that could preserve the immune cells from the primary tissue (17). Therefore, we could evaluate our PDTs and patient-derived healthy organoids (PDHOs) response to onco-virotherapy. Onco-virotherapy is based on using viral vectors that were genetically modified to present oncolysis and immuno-stimulation properties, commonly named “oncolytic viruses”. In this context, we have assessed two oncolytic viruses. First, an oncolytic vaccinia virus (VACV) that was engineered to express GM-CSF, a cytokine that favors the induction of cytotoxic immune responses mediated by T lymphocytes (18, 19). Another oncolytic virus, that doesn’t encode for GM-CSF, was used as a control (20). We will refer to VACV for the control, and VACV GM-CSF+ along the article. We assessed both oncolytic viruses for their ability to induce viral oncolysis and immune cells responses in our *in vitro* patient-derived model. To investigate the immune cell pathways, we used proteomics to help decipher proteins involved in promoting immune responses at the level of individual patients.

## 2 Methods

### 2.1 Lung tumor and healthy tissue engineering

**Figure 1:**
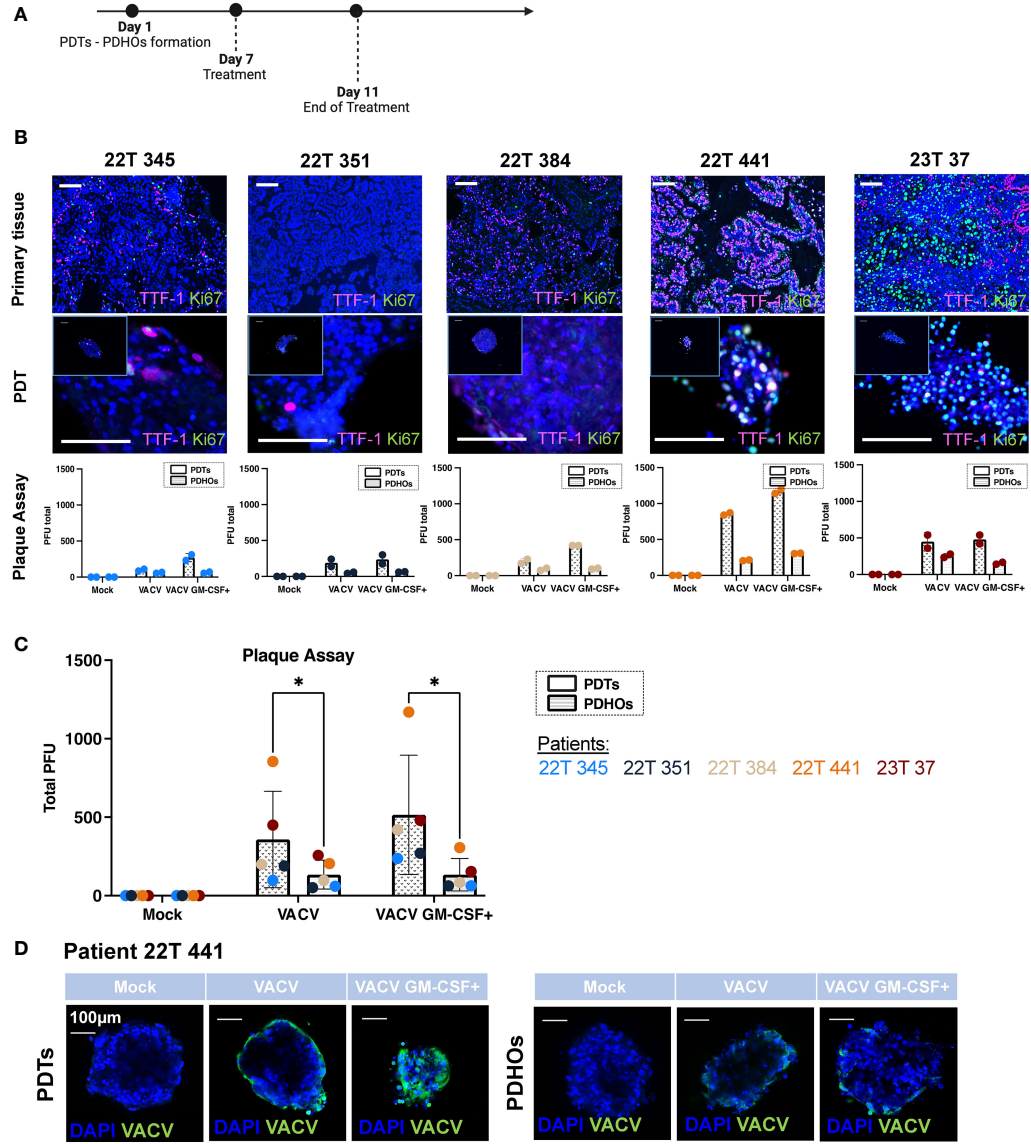
Oncolytic viruses VACV and VACV GM-CSF+ present tumor specificity and are dependent of tumoral cells' metabolic activity. **(A)** Workflow of PDTs and PDHOs oncolytic viruses' treatment. **(B)** IF of TTF-I and Ki67 biomarkers on primary tissue and PDT's paraffin slides. Scale bar 100μm. Quantification of viral particles within PDTs and PDHOs with the plaque assay based on the individual experiments (in duplicate). **(C)** Quantification of viral particles within PDTs and PDHOs with the plaque assay based on the average of technical values. P-values were calculated using an unpaired t-test with Holm-Šídák method. *p < 0,05. **(D)** IF of anti-vaccinia virus (VACV) on PDTs and PDHOs infected with VACV and VACV GM-CSF+ (e.g., patient 22T 441). Scale bar 100μm.

Human specimens were obtained by surgical resections at Hôpitaux Universitaires de Strasbourg, France. Their tumoral status was confirmed by anatomopathological analysis. The anatomopathologist confirmed the presence of a tumor on mirror samples based on morphology studies with a hematoxylin & eosin staining (**Supp. Data Sheet Figure 1**). Healthy lung tissue samples were obtained from the peri-tumoral tissue of the same donor. Patients’ informed consents were managed by the Centre de Ressources Biologiques (CRB). For information, lung cancer patients that did not present a driver mutation were selected for this study.

After the reception, human specimens were washed in PBS to remove blood excess, then enzymatically digested using the tumor Dissociation Kit, Human (ref. 130-095-929, Miltenyi Biotech) with the gentleMACS™ Octo dissociator with heaters. This kit was optimized for facilitating the maintenance of tumor-infiltrating lymphocytes while preserving important cell surface epitopes. The content of the C tube (ref. 130-093-237, Miltenyi Biotech) is filtered through a 70µm cell strainers. Strained cells were centrifuged, and pellets were resuspended in 1mL lysis buffer (ref. R7757-100mL, Sigma) to remove the remaining blood cells. A second centrifugation was performed, and the left cells were resuspended in 1mL DMEM-F12 (ref. BE12719F, Lonza).

### 2.2 Generation of patient-derived tumoroids and healthy organoids

As described before in our previous studies, for the formation of patient-derived tumoroids (PDTs) and healthy organoids (PDHOs) in a matrix-free condition, supportive cells such as adipose-tissue-derived microvessels (ad-MVs) were added (12). They were prepared according to the protocol from the supplier Advanced Solutions® and the previous studies.

PDTs and PDHOs were prepared according to a mix of 5000 patients’ cells and 5000 ad-MVs (within 1 PDT or PDHO). Cells’ suspension was then diluted in cell culture media which is composed of a mix of DMEM high glucose (ref. 41966-029, Gibco), RPMI (ref. 10101-145, Sigma) and TexMACS™ media (ref. 130-097-196, Miltenyi Biotech). This cell culture media was supplemented with different growth factors to improve patient ‘s cells *in vitro* culture, maintenance of tumor-infiltrating immune cells and ad-MVs (details in **Table 1**).

**Table 1:** PDTs and PDHOs culture media composition.

Product	References	Final concentration
Fetal Bovine Serum	10101-145, Sigma	10% of the final volume
Gentamycin	G1272, Sigma	1% of the final volume
B27 50X	17504-044	1X
VEGF-165	H9166-10µg	50ng/mL
HGF	SRP6014-10µg	30ng/mL
FGF-2	130-093-839-10µg	20ng/mL
EGF	GF316-500µg	100ng/mL
IL-2	130-097-744, Miltenyi Biotech	20UI/mL

After that, 200µL of this cell suspension was transferred on a ULA U bottom 96 wells plate (ref. 174929, Thermofisher) to form PDTs and PDHOs. The medium was changed every 3-4 days by replacing IL-2 with two other interleukins: IL-7 at 155UI/mL (ref. 130-095-361, Miltenyi Biotech) and IL-15 at 290UI/mL (ref. 130-095-762, Miltenyi Biotech).

### 2.3 Histology and immunohistochemistry

Primary tissue samples were fixed in 10% neutral-buffered formalin (ref. HT501128-4L, Sigma-Aldrich) and processed for histologic examination including hematoxylin and eosin (H&E) (**Supp. Data Sheet Figure 1**). 5µm thick tissue sections from selected paraffin blocks for each specimen were used for immunohistochemical analysis. PDTs and PDHOs were fixed in 4% PFA (ref. 416250397, Roti-Histofix®) for 1 hr at room temperature (RT) and embedded in Histogel specimen processing gel (ref. HG-4000-012, ThermoFisher Scientific) before dehydration in the Pearl. After paraffin inclusion, the blocks were sectioned on the Leica microtome at the thickness of 5μm. Then, IHC staining were performed on these sectioned slides using the LEICA Bond-II system with Bond Polymer Refine Detection based on the Novolink-polymer (ref. 7161, Leica), which is a horseradish peroxidase (HRP)-based polymer conjugated fluorescent dye that labels anti-rabbit antibody. The first steps of IHC are the dewaxing and the antigen unmasking (either High pH buffer or citrate buffer during 20min). Saturation of endogenous peroxydases with 10min of incubation in 3% H2O2 (ref. H1009, Sigma) and blocking step with goat serum for 30min (ref. G6767, Sigma) were performed. Primary antibodies were incubated for 1 hr at RT (**Table 2**). Then, after 1 hr of incubation with the primary antibody, secondary antibodies [or post-primary which is a rabbit anti-mouse IgG (ref. 7161, Leica)] and tertiary antibody (Novolink-polymer which is an anti-rabbit Poly-HRP) were incubated during 30 min at RT sequentially (ref. 7161, Leica). The post-primary was applied for primary mouse antibodies only. The final step was the signal amplification based on TSA using Perkin Elmer kit (ref. SAT701B, Perkin Elmer), for 10min at RT. Cell nucleus were stained with DAPI (dilution 1:10000 in PBS; ref. B2883, Sigma) during 10min at RT. The washing steps were performed between each step with Bond wash solution 1X (ref. AR9590, Leica). Images were captured using the fluorescence microscope Nikon Eclipse 90.

**Table 2:** List of primary antibodies used in immunohistochemistry assays.

Target	Host species	References	Dilution
TTF-1	Rabbit	Ab76013, Abcam	1:250
Ki-67	Rabbit	LS-B13463-100 LSBio	1:5000
CK7	Mouse	BSH-2018-100, Nordic Biosite	1:300
MUC1	Mouse	NCL-MUC1-CORE, Novocastra	1:200
CD45	Rabbit	13917S, Cell Signaling	1:250
PD-L1	Rabbit	13684, Cell Signaling	1:200

The value of dilution is based on the concentration of the antibody in the stock solution.

H&E staining was also performed on paraffin sections that were prior deparaffinized in xylene (ref. 185566, Honeywell) for 5 min twice and in different ethanol solutions (3 min twice in ethanol 100%, 5 min in ethanol 70%, and 5 min in ethanol 30%). Colorations with hematoxylin (ref. HHS-16, Sigma) for 3 min and eosin (ref. 318906, Sigma; diluted in distilled water at 1/50) for 30 sec were done. Differentiation for 2 min with ethanol 80% was done, followed by dehydration with ethanol 100% for 2 min and xylene for 2 min. Mounting was done with the Eukitt (ref. 045798, D. Dutscher).

PDTs and PDHOs were fixed for IHC staining of CD4, CD8 and CD20 in 4% PFA (ref. 416250397, Roti-Histofix®) for 1 hr at room temperature (RT) and embedded in Histogel specimen processing gel (ref. HG-4000-012, ThermoFisher Scientific) before dehydration in the Pearl. After paraffin inclusion, the blocks were sectioned on the Leica microtome at the size of 5μm. Then, IHC stainings were performed on these sectioned slides (**Supp. Data Sheet Figure 7**). IHC staining was performed on the LEICA Bond-II system using Bond Polymer Refine Detection based on the Novolink-polymer (ref. 7161, Leica), which is a horseradish peroxidase (HRP)-based polymer conjugated fluorescent dye that labels anti-rabbit antibody. The first steps of IHC are the dewaxing and the antigen unmasking (either High pH buffer or citrate buffer during 20 min). Saturation of endogenous peroxydases with 10 min of incubation in 3% H2O2 (ref. H1009, Sigma) and blocking step with goat serum for 30 min (ref. G6767, Sigma) were performed. The first primary antibody (anti-CD4) was incubated for 1 hr at RT. Then, after 1 hr of incubation with the primary antibody (**Table 3**), post-primary, a rabbit anti-mouse IgG (ref. 7161, Leica) was incubated for 30 min, and a tertiary antibody (Novolinkpolymer which is an anti-rabbit Poly-HRP) was then incubated during 30 min at RT (ref. 7161, Leica). The final step was the signal amplification based on TSA using Perkin Elmer kit (ref. FITC FP1018, Perkin Elmer), for 10 min at RT. A prior step to eliminate the first primary antibody and to inhibit the enzymes was used with the Linblock reagent (ref. RAG0149UK, Linaris). It was incubated twice for 2 min at RT, before saturation of endogenous peroxydases and blocking step with goat serum. The second primary antibody (anti-CD8) was incubated for 1 hr at RT, followed by the post-primary and tertiary antibody as described before. The following step was the signal amplification based on TSA using Perkin Elmer kit (ref. Cy5 FP1117, Perkin Elmer), for 10 min at RT. Then, the linblock reagent was used a second time to perform the staining of anti-CD20 and the protocol was the same, except that the signal amplification was based on TSA with the fluorescent dye Cy3 (ref. FP1046). At the end of the protocol, cell nuclei were stained with DAPI (dilution 1:10000 in PBS; ref. B2883, Sigma) during 10 min at RT. The washing steps were performed between each step with Bond wash solution 1X (ref. AR9590, Leica). Images were captured using the fluorescence microscope Nikon Eclipse 90.

**Table 3:** List of primary antibodies for IHC staining of immune cells.

Target	Host species	References	Dilution
CD4	Mouse	NCL-CD4-368, Novocastra	1:75
CD8	Mouse	M-7103, DAKO	1:2500
CD20	Mouse	M-0755, DAKO	1:5000

### 2.4 Preparation of oncolytic virus solutions

VACV or TG6002 is a replication-competent Copenhagen Strain vaccinia (20). The complete DNA sequence of vaccinia virus: thymidine kinase gene-inactivated, ribonucleotide reductase gene-inactivated.

VACV GM-CSF+ or JX-594 is a replication-competent Wyeth strain vaccinia, thymidine kinase gene-inactivated (18, 19).

The oncolytic viruses were amplified using chicken embryo fibroblasts and purified using tangential flow filtration (TFF). Briefly, the crude harvest containing infected cells and culture supernatant and conserved at -20°C, was thawed at room temperature and the viral suspension was homogenized using a homogenizing mixer equipped with an in-line chamber. Large cellular debris were then eliminated by depth filtration using filters of 5 µm pore size. The clarified viral suspension was subsequently concentrated and diafiltered in a formulation buffer by using filtration and 0,2 µm pore size hollow fibers. Finally, the purified virus was further concentrated using the same tangential flow filtration system and aliquoted before storage at -80°C until use.

The viral titer of the purified material was then measured using plaque assay on Vero cells (described in section 2.6.).

Oncolytic viruses were prepared according to a multiplicity of infection (MOI) of 0,1. This means that one viral particle is applied to ten cells. Mock is the condition containing only DMEM media. PDTs and PDHOs were incubated with oncolytic viruses for 96 hrs.

### 2.5 Immunofluorescence

PDTs and PDHOs were fixed in 4% PFA (ref. 416250397, Roti-Histofix®) for 24 hrs at 4°C. The day after, PDTs and PDHOs were incubated with PBS-Triton X100 0,25% (ref. X100, Sigma) during 30 min at RT then with PBS-BSA 5% (ref. A9647-100G, Sigma) during 4h at RT. Primary antibody (**Table 4**) was prepared in PBS-BSA 5% and incubated for 3 days at 4°C.

**Table 4:** Primary Antibody used in Immunofluorescence assay.

Target	Host species	References	Dilution
Vaccinia Virus	Mouse	Monoclonal DMAB4487, Creative Diagnostic	1:300

Four PBS-washings were performed at 30 min intervals (at RT), including one overnight washing at 4°C. Then, secondary antibody (**Table 5**) was diluted in PBS-BSA 5% and incubated for 24 hrs at 4°C.

**Table 5:** Secondary Antibody used in Immunofluorescence assay.

Target	Host species	References	Dilution
Anti-mouse FITC	Goat	A21121, Invitrogen	1:200

Three PBS-washings were performed at 30min intervals (at RT), and then, DAPI (ref. B-2883, Sigma; Dilution 1:2500) was added to PDTs and PDHOs for 1 hr at RT. PBS then replaced DAPI until acquisition on confocal microscopy LSM Zeiss 800.

### 2.6 Titration and infection of Vero cells

Prior to titration, supernatants, and PDTs or PDHOs were harvested in duplicate for each condition and stored at -80°C before use.

The day of titration, samples were thawed in water bath at 37°C and then refrozen in dry ice. Samples were thawed and refrozen alternatively at least three times to lyse the PDTs and PDHOs. Vero cells (ATCC CCL-81™) were split and seeded at 5.10^4^ cells/well in a six wells-plate. Cell culture media was DMEM (ref. 41966029, Gibco), supplemented with 1% gentamycin and 10% FBS. The day after, serial dilutions (E^-1^ to E^-6^) of supernatants comprising lysed PDTs and PDHOs were prepared. Dilutions were done in PBS (ref. D8537-500mL, Sigma) supplemented with 1% FBS and 1% of cations [Mg (CH_3_COO)_2_ (Cf = 10g/L) and CaCl_2_ 2(H_2_O) (Cf= 10g/L)]. These different solutions were then incubated for three days at 37°C. Solutions containing viral particles produce some lysis zones on Vero cells, which are also called viral plaques. These viral plaques are counted after three days of incubation by using the neutral red solution 10% (ref. N2889-100mL, Merck-Millipore) diluted in DMEM media with 20% agarose (Cf = 50g/L) (ref. A9045-250g, Sigma).

### 2.7 Proteomics

#### 2.7.1 Protein extraction

Organoids from the same condition were pooled (Total 6) in an Eppendorf and rinsed with PBS. The PBS was removed, and the organoids were frozen at -80°C before extraction. Radio immunoprecipitation assay (RIPA) lysis buffer (ref. 89901, ThermoScientific) supplemented with Phosstop (ref. 04 906 837 001, Roche) and cOmplete tablets (ref. 4693116001) were used to lyse the organoids under sonication to perform protein extraction. The amount of protein was quantified using the DC protein assay kit (ref. 500-0116, Bio-Rad) according to the supplier’s recommendations.

#### 2.7.2 Preparation of supernatants

Supernatants from PDTs and PDHOs, mock and infected ones, from 6 different wells were pooled in an Eppendorf. The supernatants containing onco-virotherapy treatment were filtered through a 0,1µm filter and stored at -80°C until shipment to Olink Proteomics®, Uppsala, Sweden.

#### 2.7.3 Olink proteomics

Lysates obtained from PDTs and PDHOs or supernatants (for the secretome analysis), mock and infected ones, were sent to Olink Proteomics®, Uppsala, Sweden. The panel Olink Target 96 Immuno-Oncology was performed using the proximity extension assay (PEA) technology. The assay is based on dual recognition of each protein by a pair of antibodies that link to the same protein in the samples to analyze. These antibodies are coupled with a complementary single DNA oligo strand that hybridize when in a close proximity once fixed on the protein target. The double strand is extended via a first PCR reaction to amplify the signal and generate amplicons to detect and quantify by qPCR targets. Proteins’ concentrations are normalized starting from Ct values to NPX arbitrary units on an inverted log 2 scale. This normalized protein expression (NPX) is obtained by subtracting the Ct values obtained for each protein from a control Ct value constituted by the extension control, spiked in the same concentration in each well. First, the Ct value of the extension control, for a single sample, is subtracted from the Ct value of each analyte tested in a panel. This results in a delta Ct. This first step adjusts for any well-to-well technical variation. In the second step, the data is related to a known standard by subtracting the median IPC (Inter Plate Controls) Ct values of an analyte from the delta Ct of the same analyte, producing the delta delta Ct. This step here reduces inter plate variation. And finally, a correction factor determined during the validation of the Target 96 kit is used to invert the scale. The delta delta Ct for each analyte is subtracted from the Correction factor which then generates the NPX values. This inversion makes the NPX values more intuitive for data interpretation.

#### 2.7.4 Bioinformatics analyses

Data were analyzed using the R-script and the R package of OlinkAnalyze.

KEGG Analysis: Gene set enrichment analyses (GSEA) were performed with the function olink_pathway_enrichment from OlinkAnalyze package using Kegg pathways. 

#### 2.7.5 Proteins' level measurements in lysates

GM-CSF (ref PPX-03-MXKA49V) was analyzed via Procarta Plex technology. Procarta Plex designed this kit using magnetic bead technology. Experimental steps were performed according to the supplier protocol. Data was analyzed using GraphPad Prims 9. The statistical method used was a paired two tailed Student's t-test to determine changes in GM-CSF proteins under onco-virotherapy treatment. n represents the number of patients used for each assay and a significance threshold of p<0,05 was used to determine a significant effect of onco-virotherapy treatment.

## 3 Results

### 3.1 Oncolytic viruses VACV and VACV GM-CSF+ present tumor specificity and are dependent of tumoral cells’ metabolic activity

**Figure 2:**
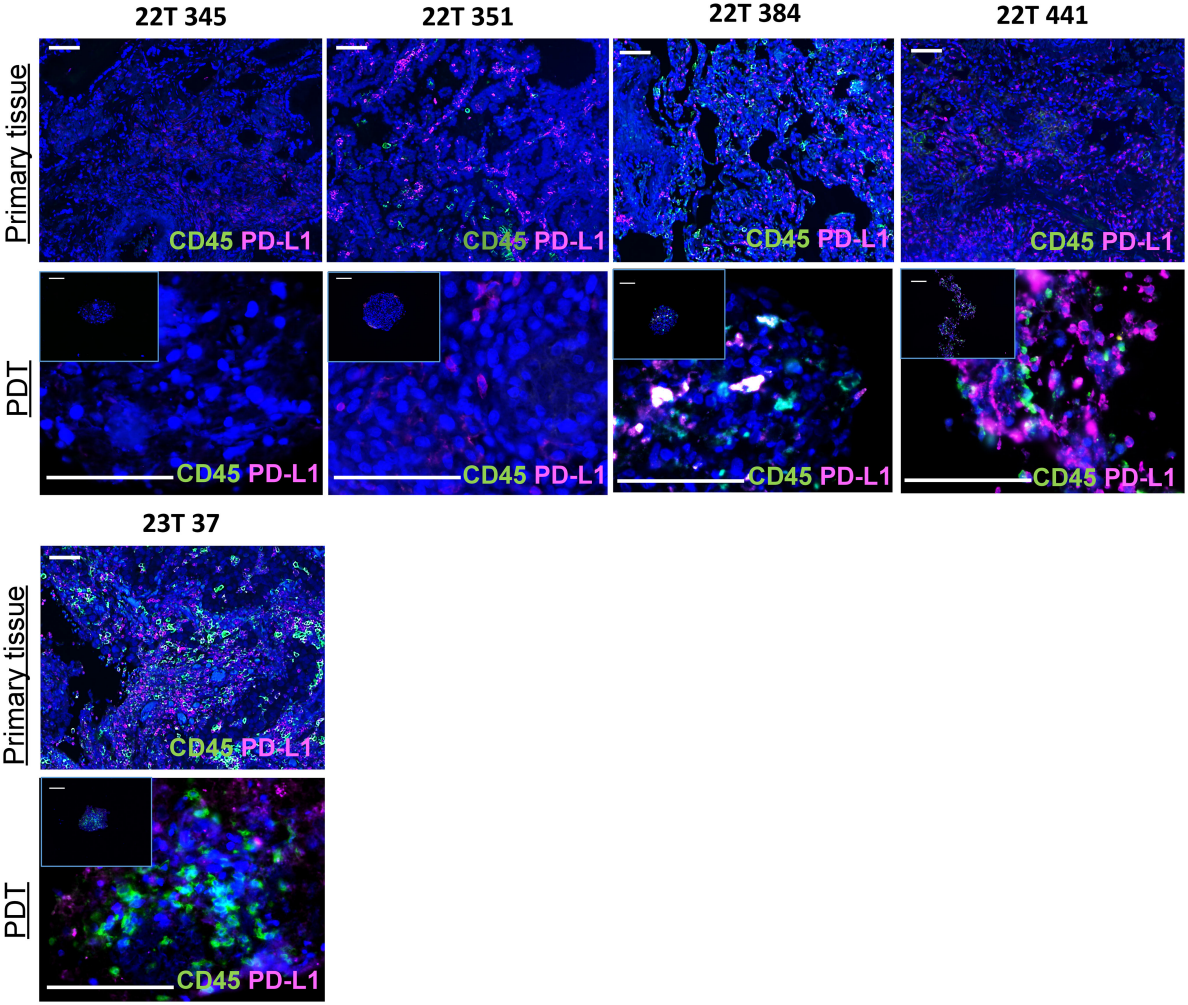
An immunocompetent model of PDTs and PDHOs to evaluate immunostimulatory properties of oncolytic viruses. Biomarkers CD45 and PD-L1 expression were analyzed on patients' primary tissues and patients' derived tumoroids. Scale bar 100μm.

**Figure 3:**
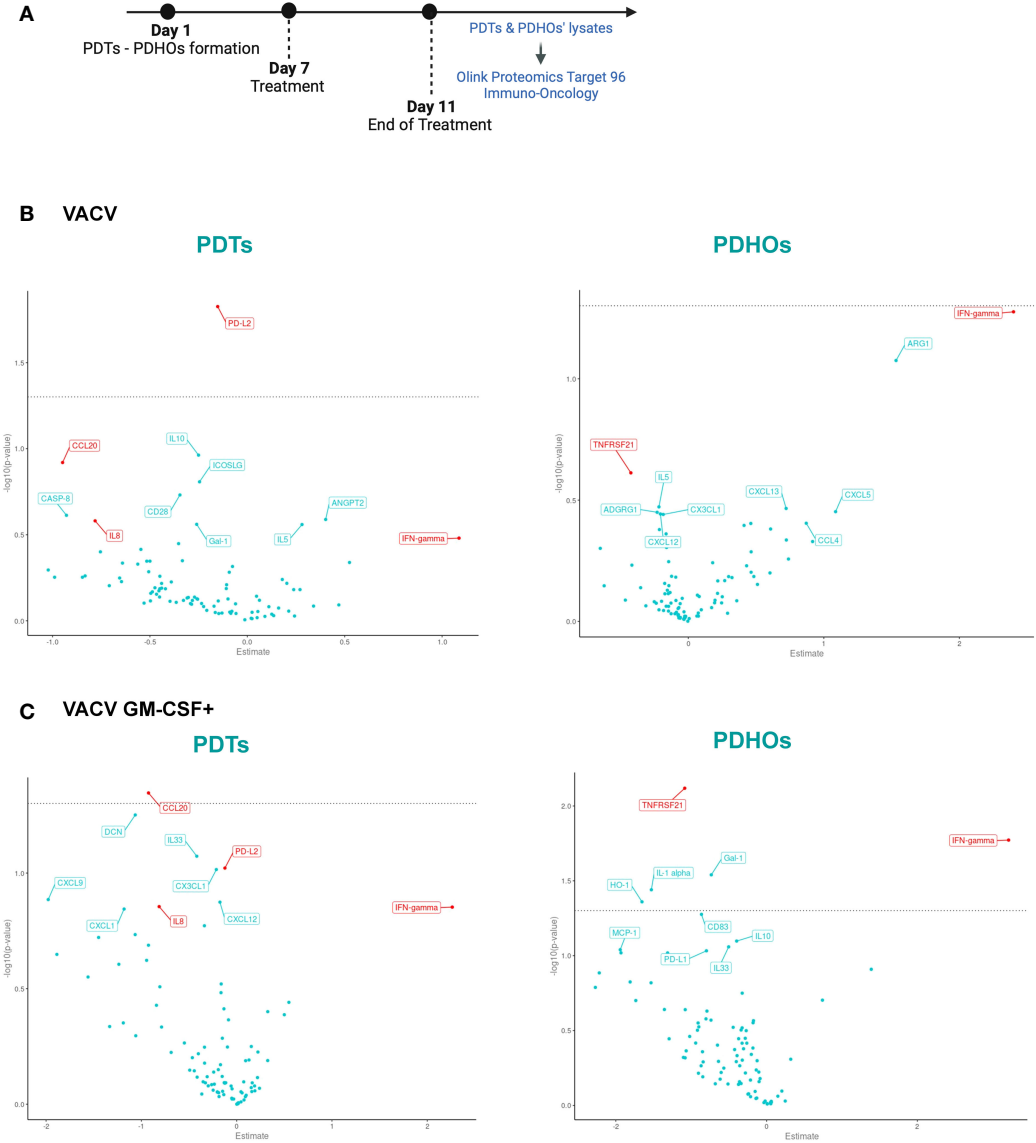
Volcano Plot showed some downregulation of proteins involved in tumoral progression in PDTs. **(A)** Workflow of PDTs and PDHO to proteomic' analysis. **(B)** Volcano plots of differentially expressed proteins are shown for the VACV versus mock n=5. **(C)** Volcano plots of differentially expressed proteins are shown for the VACV GM-CSF+ versus mock n=5 (red genes: proteins found in top 10 upon VACV and VACV GM-CSF+ treatment in either healthy or tumoral samples).

**Figure 4:**
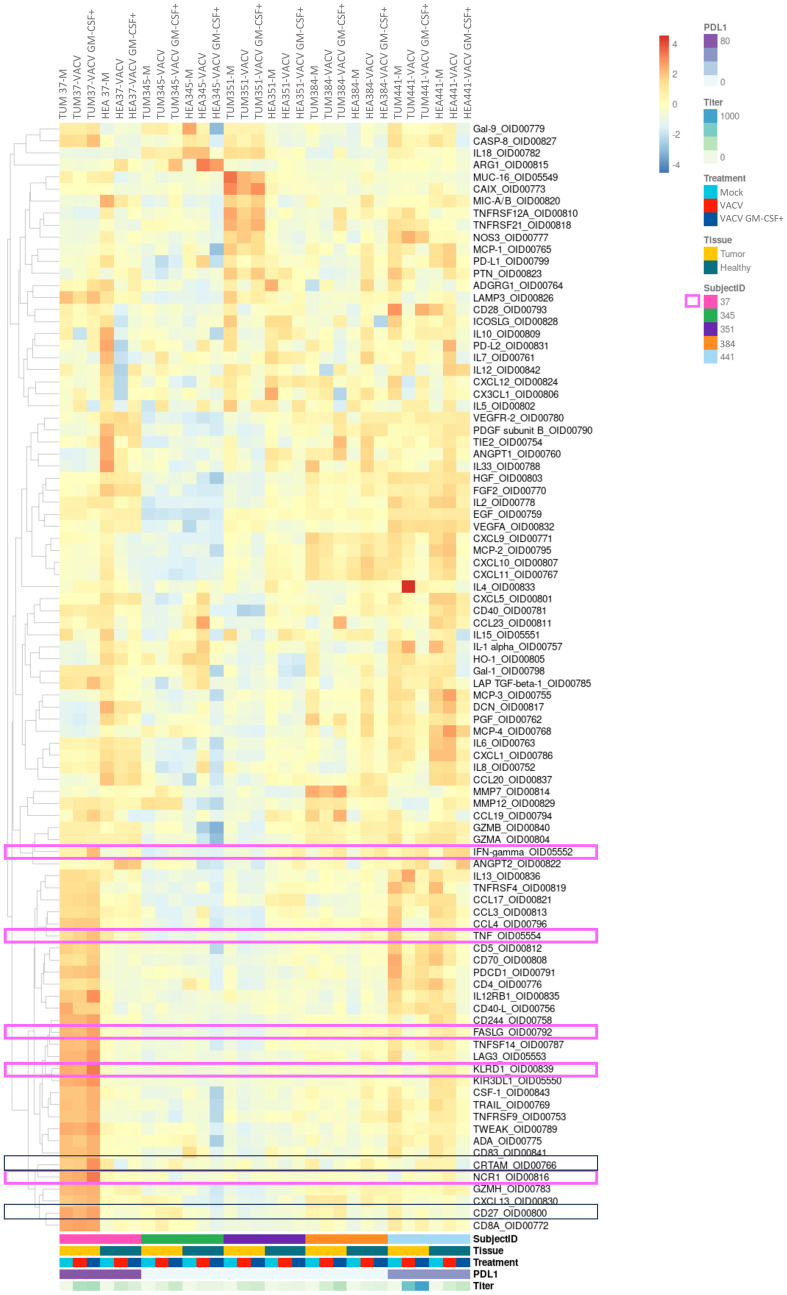
Analysis of intracellular proteins showed some immunomodulatory effects of oncolytic viruses in PDTs and PDHOs. (Heatmap showing the expression of detectable proteins based on a z-score and on a clustering cluster_rows=FALSE.) n=5.

In our study, we generated PDTs and PDHOs from lung adenocarcinoma and the healthy counterpart of the tumor respectively (refer Method section: lung tumor and healthy tissue engineering). They were cultured according to the methods described in previous research (12). PDTs and PDHOs were infected with a multiplicity of infection (MOI) of 0,1 to evaluate the permissivity of patient’ s cells to oncolytic viruses, for 96 hrs (**Figure 1A**). We assessed main lung adenocarcinoma marker, TTF-1 (21) on primary tumor tissues (**Figure 1B**) and primary healthy tissue (**Supp. Data Sheet Figure 2**), then verified its expression on respective PDTs and PDHOs. Other main biomarkers of lung adenocarcinoma such as cytokeratin 7 (CK7) and mucin-1 (MUC1) were also assessed by IHC on PDTs and PDHOs. Globally, PDTs could maintain the expression of these three biomarkers, and more cells were stained in PDTs than in PDHOs (**Supp. Data Sheet Figures 3**, **4**).

The biomarker Ki-67 defines a rapid cell division and high metabolic activity; we then evaluated the biomarker Ki-67 in concomitance with TTF-1. We can observe that patients 22T 441, and 23T 37 (both in stages IIB) were the ones presenting more Ki-67 expression in primary tumor tissues and PDTs (**Supplementary Table 1**; **Figure 1B**). The quantification of viral particles produced in cells was evaluated with the plaque assay. We can observe that the number of viral particles increases when the biomarker Ki-67 is predominant generally (**Figure 1C**). This biomarker was more expressed for patients 22T 441 and 23T 37.

Furthermore, the number of viral particles was more important in PDTs than in PDHOs. This observation is consistent as we observe low expression of Ki-67 in healthy primary tissues and matched PDHOs (**Supp. Data Sheet Figure 2**).

The presence of vaccinia virus particles can be further confirmed with immunofluorescence using an antibody targeting vaccinia virus (**Figure 1D**; **Supp. Data Sheet Figure 5**). For organoids from patient 22T 441, we could observe a positive staining for vaccinia virus with both oncolytic viruses. On PDTs, VACV infected on the periphery whereas VACV GM-CSF+ infected cells with more spreading (**Figure 1D**). On PDHOs, VACV and VACV GM-CSF+ infected fewer cells compared to PDTs. This observation supports onco-virotherapy specificity for tumoral cells.

Viral replication is known to be supported by the host cellular metabolic state within the TME. We could point out that not all tumor-derived cells were metabolically active. Few co-staining of biomarkers Ki-67 and TTF-1 was observed in both type of samples. As both oncolytic viruses were deleted for enzymes involved in VACV DNA synthesis, this makes them rely heavily on the pool of these enzymes present in the target tumoral cells to support their proliferation (20). Contrary to what we might think, not all live tumoral cells are highly proliferative. These low metabolic states and quiescent states were already described in solid tumors (22). And this lack of metabolic activity could hinder oncolytic viruses’ efficiency. In this part, results supported specific replication of both oncolytic viruses within PDTs.

### 3.2 An immunocompetent model of PDTs and PDHOs to evaluate immunostimulatory properties of oncolytic viruses

Prior to performing proteomics to evaluate immunostimulatory properties of oncolytic viruses, we checked the maintenance of primary tumor immune cells within PDTs and PDHOs. We have optimized the cell culture media to maintain them along the culture (**Figure 2**; **Supp. Data Sheet Figures 6**, **7**). These immune cell subpopulations from the primary tissue are likely to have a better predictive value of prognosis and therapeutic responses (23). Maintaining these tumor-infiltrating immune cells in our 3D model was more relevant to reflect the tumor immune microenvironment better. We used the biomarker CD45 to assess the maintenance of tumor-infiltrating immune cells within PDTs, PDHOs, and their corresponding primary tissues (**Figure 2**; **Supp. Data Sheet Figures 6**, **7**). We could observe CD45 biomarker expression in both type of samples (tumor tissues and PDTs). Two patients 22T 384 and 23T 37 presented more immune cells in their primary tumoral tissues, and this was also reflected on their PDTs. Then, healthy primary tissues presented fewer immune cells compared to the tumoral primary tissue. This was also observed on PDHOs compared to PDTs (**Supp. Data Sheet Figure 6**). As for patients 22T 345 and 22T 351, no CD45 expression was observed in corresponding PDTs. In patient 22T 351, we even observed a loss of CD45 expression, this could be due to the difficulty of maintaining immune cells *in vitro*.

Immune or tumoral cells can express PD-L1 to reduce antitumor immunity and inhibit T cell activation. The knowledge of PD-L1 tumoral cells expression is important as this ligand has emerged as a potential biomarker to predict responses to immunotherapy (24, 25). Then, we assessed PD-L1 biomarker on PDTs as well and, we could find the expression of PD-L1 in PDTs (**Figure 2**). When there’s no colocalization of PD-L1 with CD45, we suggest that it is expressed by tumoral cells. This was the case for all patients except patient 22T 345 whose PD-L1 expression is 0%. In contrast, PDTs from patient 22T 441 presented more PD-L1 expression, which is up to 60% in clinical features (**Supplementary Table 1**).

These PDTs and PDHOs could maintain immune cells from the primary tissue. Hence, from these PDT and PDHO model, we could assess immune-related effects following oncolytic viruses’ treatment.

### 3.3 Analysis of intracellular proteins with multiplex protein screening showed downregulation of proteins involved in tumoral progression in PDTs

The use of genomics on fresh tumor tissues or formalin-fixed paraffin-embedded (FFPE) blocks has permitted to have insights on genetic variants that predict responses to drugs (26, 27). The cancer genome can be completed by having a look on the protein products from these genes (intracellular and membranous proteins) (28). These proteins represent most of the human proteome besides secreted proteins. They are the molecules mostly responsible for cell growth and cancer progression (28). To better understand oncolytic viruses’ effect at the protein level, we measured proteins relative expression with the proximity extension assay (PEA) technology. This technology is supported by the Olink platform (Olink Target 96 Immuno-Oncology). We mainly investigated the immune responses at the intracellular proteins (**Figure 3A**).

To investigate oncolytic viruses’ global effect on the five patients, specifically selected for their different histology, proteomic data can be summarized under a Volcano Plot representation (**Figures 3B, C**). This data representation permits an overview of differentially expressed proteins on PDTs and PDHOs, either treated by VACV or VACV GM-CSF+. After both oncolytic viruses’ treatment, levels of IFN-γ were upregulated (**Figures 3B, C**). This upregulation is a sign of antiviral responses in the TME (29). Then, both oncolytic viruses mainly presented a downregulation of proteins involved in tumoral cell progression and survival, such as interleukin-8 (IL-8), C-C motif chemokine ligand 20 (CCL20), and galectin-1 (Gal-1) (**Figures 3B, C**). IL-8 (30) is a chemokine known for its pro-inflammatory and pro-angiogenic effects in NSCLC. CCL20 (31) is an oncogenic chemokine favoring tumoral progression, and Gal-1 (32) is known to favor adhesion, proliferation, and metastatic processes of tumoral cells. On PDTs, VACV GM-CSF+ could induce downregulation of other proteins involved in tumoral progression, such as CX3CL1 and CXCL12 chemokines. Regarding the effect of oncolytic viruses on PDHOs, we could note a downregulation of the TNFRSF21 (33) protein, mainly involved in the negative regulation of T lymphocytes (**Figures 3B, C**). Besides the downregulation of pro-tumorigenic effects, oncolytic viruses can elicit immune responses.

### 3.4 Analysis of intracellular proteins with multiplex protein screening showed immunostimulatory effects in PDTs

To assess the global effect of viral infection on patients’ PDTs, a gene set enrichment analysis using KEGG pathways was also done to study the pathway significantly enriched in our comparisons infected versus mock (**Supplementary Table 2**, **Supp. Data Sheet Figure 8**). The main pathway enriched by both oncolytic viruses was the chemokines pathway. Among the enriched chemokines, we can note the decrease of CCL20 expression observed previously and CCL4 (34) and that promote cancer progression. We can also note a decrease expression of chemokine implied in decreased activity of CD8+ T cells such as CXCL5 (35) by both oncolytic viruses (**Supp. Data Sheet Figure 8**).

The heatmap permits to cluster the proteins’ levels by the nature of the samples (tumor or healthy), patients’ individuality and treatments’ conditions. The proteins’ levels were defined according to the normalized protein expression values (NPX) on a log_2_ scale. Oncolytic viruses’ effects varied from one patient to another.

If looked into details, PDTs derived from patients 22T 345 and 22T 351 showed some lower protein z-score than other patients. This observation was following clinical features which are a low PD-L1 expression and the absence of CD45 expression (**Supplementary Table 1**). These features reflect a cold tumor (36) (**Figure 2**; **Supplementary Table 1**). Patient 22T 345 presented a slight immunomodulatory effect under VACV and VACV GM-CSF+ (**Figure 4**). Indeed, we could observe that the CD27 (37) protein was 1,5 to 2-fold higher in PDTs treated with both oncolytic viruses.

In contrast with these two latter patients, PDTs derived from patients 23T 37 and 22T 441 could demonstrate some immunomodulatory responses, mostly under VACV GM-CSF+ treatment. We will describe each case individually and briefly. In PDTs from patient 22T 441, we could observe an increase of IFN-γ (38), which is also a cytokine mediated by cytotoxic T cells in addition to be linked to antiviral responses. As for patient 23T 37, we observed more immune-mediated cytotoxicity effect. Indeed, we observed that proteins involved in NK-cell mediated cytotoxicity were 2-fold higher in PDTs VACV GM-CSF+ treated, with the increase of proteins Natural Cytotoxicity Triggering Receptor (NCR1) (39) that mediates MHC non-restricted cytotoxicity, and Killer cell immunoglobulin-like receptor (KIR3DL1) (40) which is the ligand for the surface protein KLRD1, involved in NK cell signaling. We can also note a slight increase of IFN-γ (38), TNF-α (41) and FASLG that participate to effector functions of NK and T lymphocytes. Then, we could observe a slight increase of Cytotoxic and Regulatory T cells Molecule (CRTAM) (42) protein involved in CD8+ T cells response (**Figure 4**; **Supp. Data Sheet Figure 8**). In summary, this patient has more response to oncolytic virotherapy. This patient presented a PD-L1 expression > 90% regarding its clinical criteria. As mentioned previously, PD-L1 expression level is among the biomarkers to define a response to immunotherapy. This suggests to integrate PD-L1 expression with tumor immune cells infiltrate to refine onco-virotherapy selection for patients (43) (**Figure 4**, framed in pink). Indeed, PD-L1 expression and a high T-cell infiltration are part of features to define a “hot” tumor which is mostly effective to immunotherapies (44).

In general, VACV GM-CSF+ presented more immunomodulatory effects than VACV mainly because of the GM-CSF transgene which might have potentiated an antitumoral immune response. The encoded transgene GM-CSF was mostly released in PDTs infected by VACV GM-CSF+ than in VACV (**Supp. Data Sheet Figure 9**).

As the research for biomarkers often relies on the secreted proteins, we also studied the proteomic signature in the secretome of two other patients 22T 31 and 22T 67 according to the same treatment workflow (**Supplementary Table 3**, **Supp. Data Sheet Figure 10A**). Indeed, biological fluids such as blood samples are used to predict outcomes on patients under ICIs treatment (45). Clinical biomarkers such as TTF-1, Ki-67, CD45 and PD-L1 were assessed in the same way as other patients (**Supp. Data Sheet Figures 10B, C**). Our experimental design permits us to establish the signature of secreted proteins after VACV and VACV GM-CSF+ treatments under a heatmap representation (**Supp. Data Sheet Figure 10D**). From PDTs of patient 22T 31, we can note that more change was seen at the level of secreted proteins. Due to its higher clinical relevance, we analyzed in greater detail the secreted proteins under VACV GM-CSF+ infection. We could observe the increase of some proteins involved in T cells activation such as CD28 (46), CD40L (47), CD70 (37), CD244 (48), CRTAM (42) and IL12 receptor subunit beta 1 (IL12RB1) (49). Also, NK-cell mediated cytotoxicity was observed with the increase of proteins NCR1 (39), KIR3DL1 (40), and Killer cell lectin-like receptor subfamily D member 1 (KLRD1) (50) or CD94 which is a surface protein involved in NK cell signaling (**Supp. Data Sheet Figure 10D**, proteins marked with a pink star). PDTs from patient 22T 67 showed less response (similarly to the intracellular proteins’ signature), we only observed a slight increase of IL2 (51), IL5 (52), and KIR3DL1 (40) (**Supp. Data Sheet Figure 10D**, proteins marked with a green star). Interestingly, we also observed a decrease of lymphocyte-activation gene 3 (LAG3) protein (53), which is involved in the TCR inhibition of T cells. Its inhibition is beneficial to an anti-tumoral effect. Overall, different profiles of drug sensitivity were observed; this could be explained with patients’ heterogeneity which is a hurdle to drugs efficiency (54).

As observed with the intracellular proteins, the immunomodulatory response induced by oncolytic viruses, especially VACV GM-CSF+, was also reflected with the secreted proteins. This demonstrates the relevance of combining the study of secreted and intracellular proteins to map the expression of the proteins under treatment. Thus, we have shown the usefulness of proteomics with tumoral organoids to bring more insights into onco-virotherapy responses. The use of proteomics gives multiple readouts in protein profiling and permits to stratify responders from non-responders.

## 4 Discussion

We have demonstrated how to maintain main features of a tumor niche, including tumoral cells and tumor-infiltrating immune cells within our PDTs and PDHOs models. Under our experimental protocol, we observed that our PDTs composition was reliable to tissues’ one. However, for some patients (e.g. 22T 351), we could observe some discrepancies between the primary tissue and matched PDTs. The loss of CD45 expression in PDTs compared to primary tissue could be explained by the difficulty of maintaining immune cells *in vitro* (15). Furthermore, differences between the organoids and primary tissues have already been described before, this could be due to the cell culture conditions and the *in vitro* growth of primary cells (55). In parallel to PDTs, we also generated PDHOs from the healthy counterpart of the tumor to assess safety-related issues. We have confirmed their non-malignancy phenotype by histology with the decreased expression of lung adenocarcinoma-associated biomarkers. Results as a whole show the ability of tumoroids and organoids to represent a potential tool for drug discovery.

The great asset of oncolytic viruses is their tumor-specificity effect (56). Then, the integration of healthy organoids was relevant to confirm their tumor-specificity and safety profile. Currently, most of the data regarding oncolytic viruses’ efficiency comes from murine models and clinical data. These data mainly concentrated on the therapy’s final effect, which was mostly the tumor site’s regression, however, this input could not add significant insight into biological responses. To increase the readouts, PDTs and PDHOs permit studying oncolytic viruses’ permissivity into human tumor cells in complementary to animal models. We could study the conceptual mechanisms of oncolytic viruses, including infection, replication ability, immune cell response activation, and GM-CSF transgene delivery. Unfortunately, our findings didn’t reach statistical significance due to following reasons: (i) the small number of patients samples, (ii) the substantial inactivation of genes implied in DNA synthesis in the genome of oncolytic viruses (making them dependent on tumoral cells), and (iii) a hot tumor seems to be a predisposing factor of onco-virotherapy efficiency.

Indeed, we have studied the oncolytic viruses’ effect on a subset of seven patients (five on lysates and two on supernatants). The construct of the oncolytic virus’s genome had an impact as VACV GM-CSF+ presented more immunomodulatory effect than VACV. One primary mechanism of Transgene’s attenuated oncolytic viruses is to replicate precisely in metabolically active cells. Our results show that not all tumoral cells, positive for lung-adenocarcinoma markers, expressed Ki-67. Moreover, it has been reported that 80% of tumoral cells are in a quiescent state within the tumor (22). We also noticed that a high PD-L1 expression (90-100%) is beneficial to onco-virotherapy efficiency. Indeed, in most of the patients analyzed, especially for patient 23T 37, we could observe a slight increase of proteins involved in NK and T cells activation including NCR1, KIR3DL1, CD27, CRTAM, FASLG, IFN-γ and CXCL13.

Altogether, there is a question of balance between the payload of oncolytic viruses, the patients immunophenotype and tumoral cells metabolic activity among other parameters (56). Based on these observations, we have a trend of patients’ profile of responders to onco-virotherapy. Thus, we suggest screening tumoral cells’ metabolic activity using the biomarker Ki-67 associated to a seahorse analysis for example. Also, the assessment of PD-L1 expression and immune-inflamed tumor using spatial transcriptomic before oncolytic viruses’ administration at clinical phases can help to predict oncovirotherapy’ effect. Next steps will be to consolidate our studies toward the immune compartment of our 3D model by studying the maintenance of native infiltrating immune cells in matched PDTs using single-cell sequencing as Neal et al. (57). did.

Once well defined, PDTs and PDHOs could be applied to study the impact of other payloads, such as cytokines and chemokines with a profile that leads to Th1-type immune response mediated by NK, CD4+, and CD8+ T cells (58). These observations can be further complemented on more complex ex vivo models such as patient-derived explants (59), and animal models, to strengthen the use of tumoroids in drug discovery.

## Data availability statement

The original contributions presented in the study are included in the article/**Supplementary Material**, further inquiries can be directed to the corresponding author/s.

## Ethics statement

The studies involving humans were approved by Ethics Committee of Grand Est, France. The studies were conducted in accordance with the local legislation and institutional requirements. The participants provided their written informed consent to participate in this study.

## Author contributions

HL: Formal analysis, Methodology, Writing – original draft, Writing – review & editing. JD: Formal analysis, Software, Visualization, Writing – review & editing. PC: Formal analysis, Methodology, Writing – review & editing. AL: Methodology, Writing – review & editing. GH: Writing – review & editing. VL: Resources, Writing – review & editing. SJ: Formal analysis, Methodology, Writing – review & editing. JB: Conceptualization, Methodology, Writing – review & editing. NB: Conceptualization, Methodology, Supervision, Writing – review & editing. EQ: Conceptualization, Supervision, Writing – review & editing.
