# Supplementary material for: Patient-derived tumoroids and proteomic signatures: tools for early drug discovery
Source: Front Immunol. 2024 Apr 18;15:1379613. doi: 10.3389/fimmu.2024.1379613 (PMC11063793; doi:10.3389/fimmu.2024.1379613)
Supplement: Supplementary file 1 [file DataSheet_1.pdf]

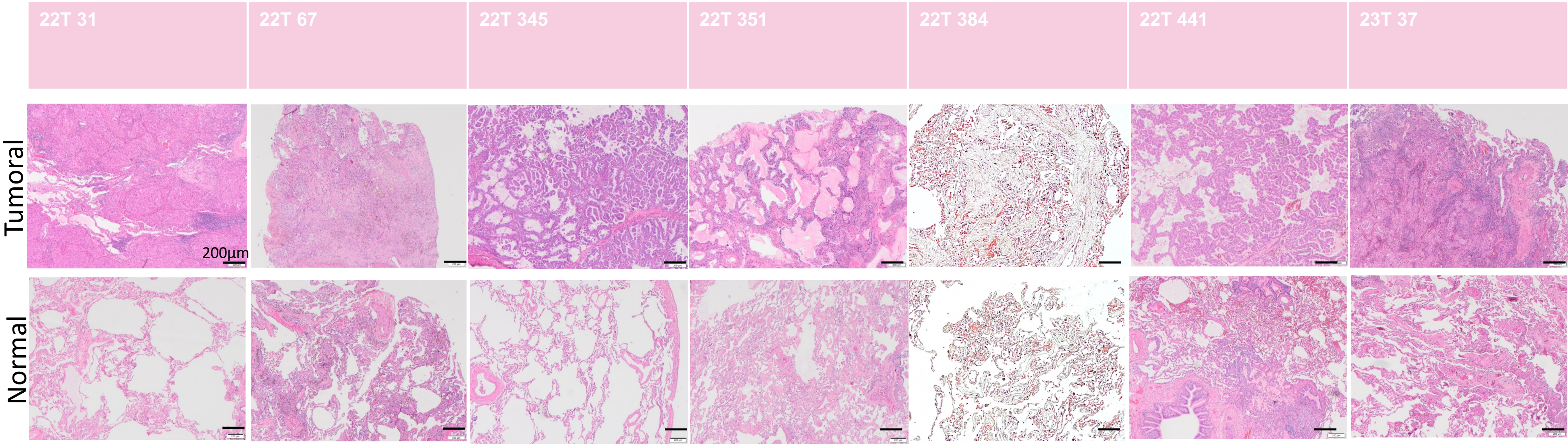

Supplementary Figure 1. Related to materials & methods. H&E colorations performed by the anatomopathologists to confirm the tumoral status of the surgical piece on tumoral tissue paraffin sections and normal tissue paraffin sections. Scale bar 200µm

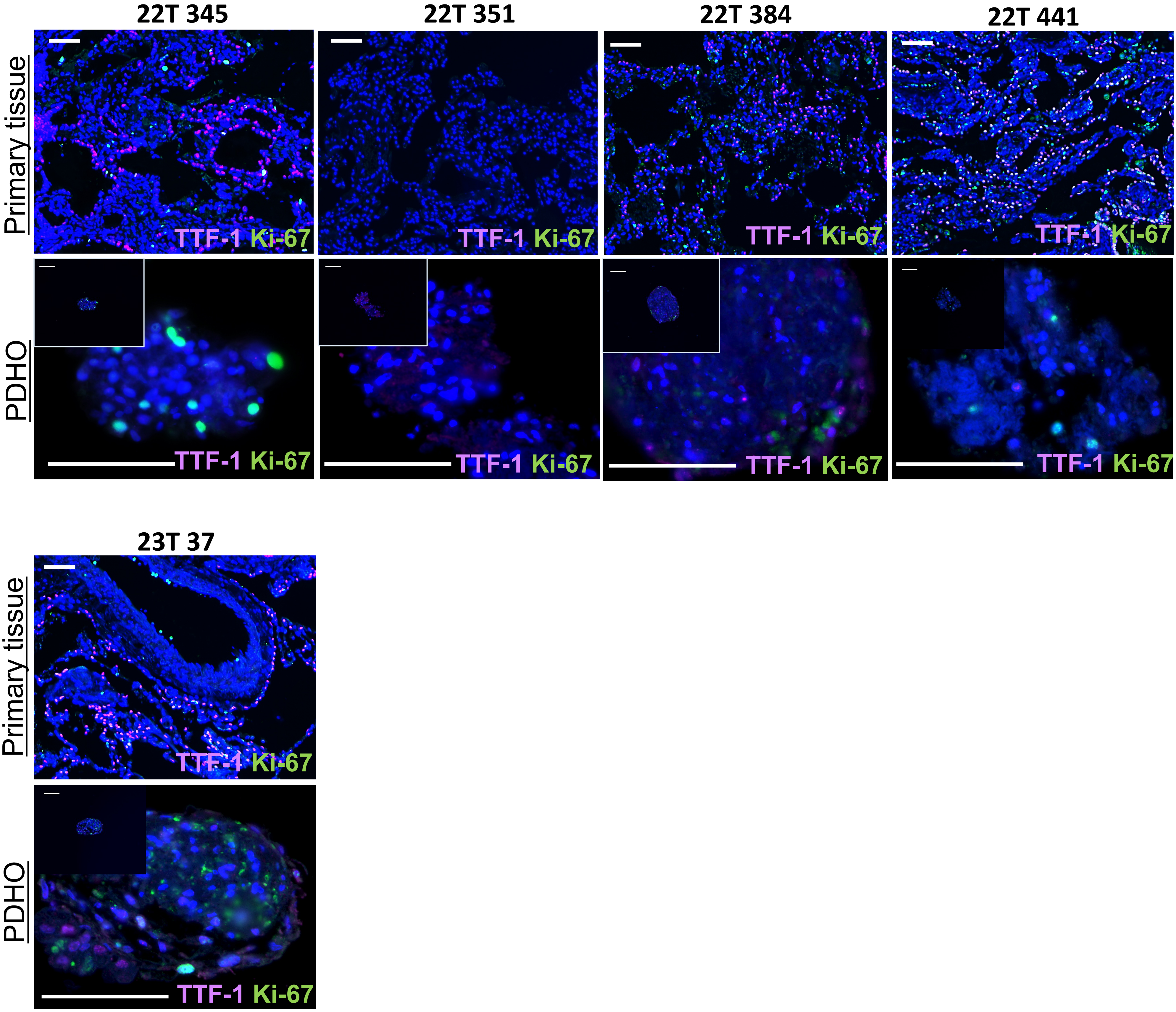

Supplementary Figure 2. Related to Figure 1. Biomarkers TTF-1 and Ki-67 expression were analyzed on patients' primary tissues and PDHOs using IHC. Scale bar 100µm

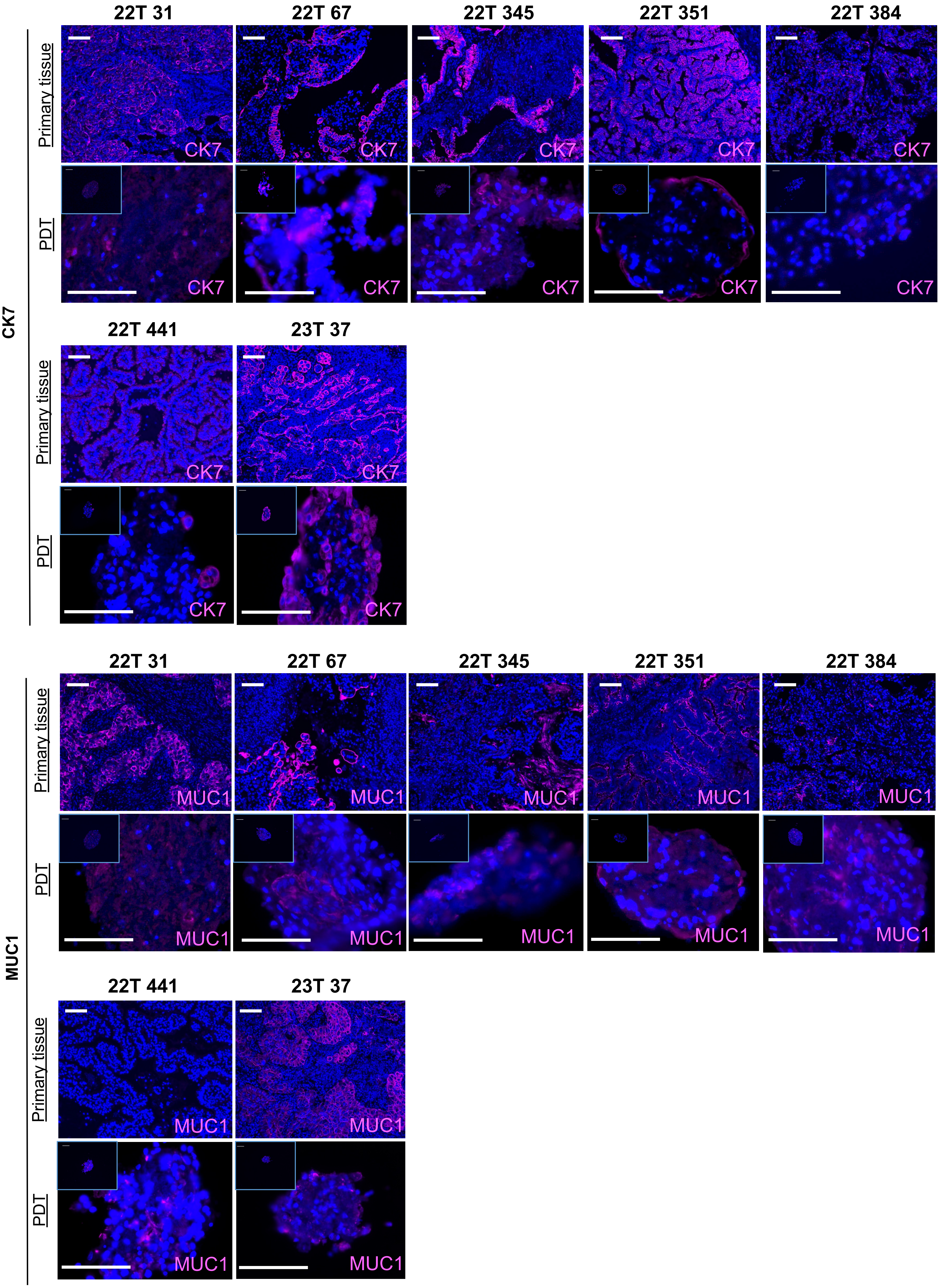

Supplementary Figure 3. Related to Figure 1. Other main biomarkers of lung-adenocarcinoma were analyzed on patient-derived tumoroids by IHC such as CK7 and MUC1. Scale bar 100μm

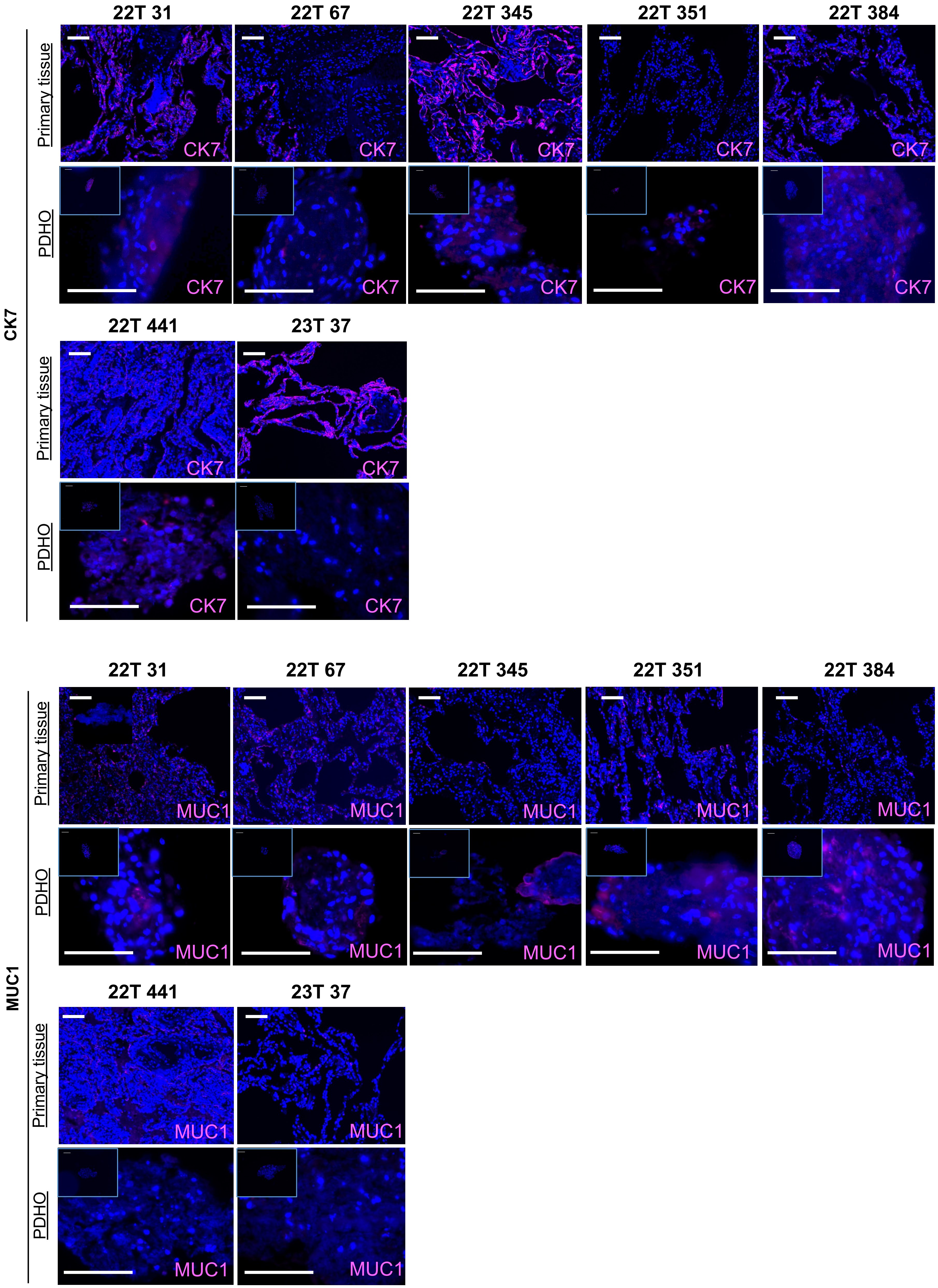

Supplementary Figure 4. Related to figure 1. Other main biomarkers of lung-adenocarcinoma were analyzed on patient-derived healthy organoids by IHC such as CK7 and MUC1**.** Scale bar 100µm

A

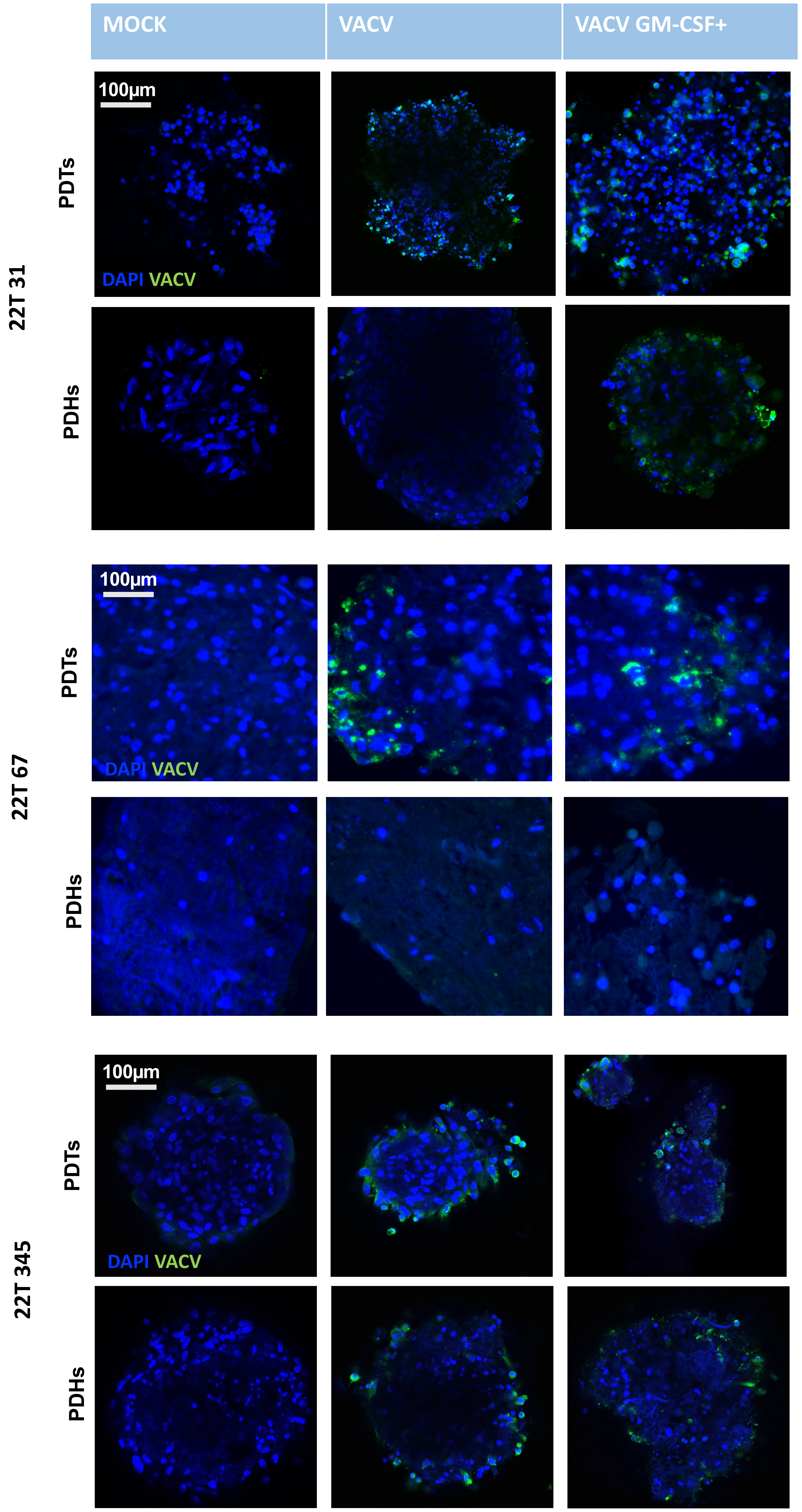

Supplementary Figure 5. Related to Figure 1. A. IF of anti-vaccinia virus (VACV) on PDTs and PDHOs infected with VACV and VACV GM-CSF + on patients 22T 31, 22T 67 and 22T 345. Scale bar 100µm

B

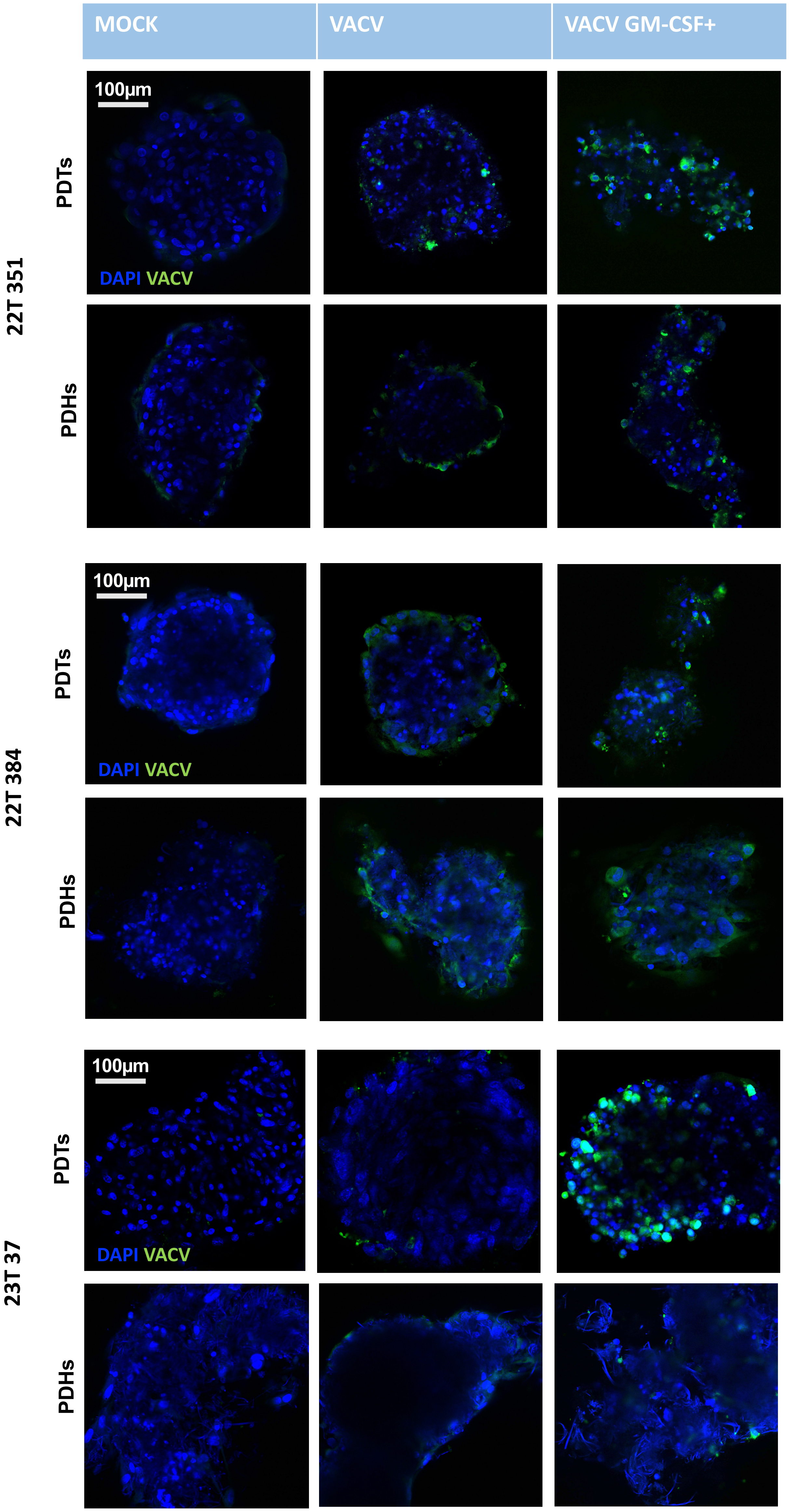

Supplementary Figure 5. Related to Figure 1. B. IF of anti-vaccinia virus (VACV) on PDTs and PDHOs infected with VACV and VACV GM-CSF + on patients 22T 351, 22T 384 and 23T 37. Scale bar 100µm

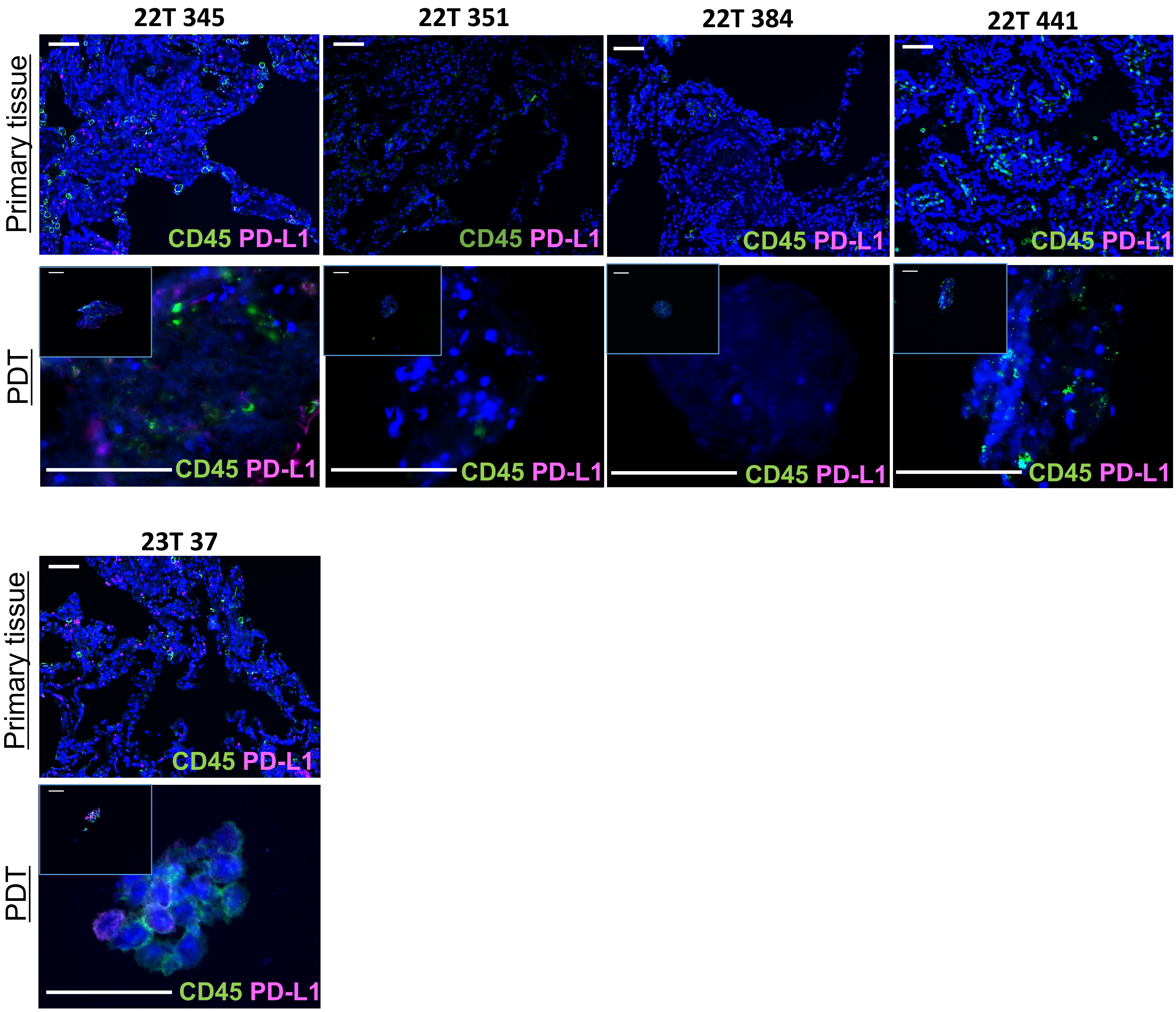

Supplementary Figure 6. Related to Figure 2. An immunocompetent patient-derived organoid model. Biomarkers CD45 and PD-L1 expression were analyzed on patients' primary tissues and patients' derived 3D models using IHC. Scale bar 100µm

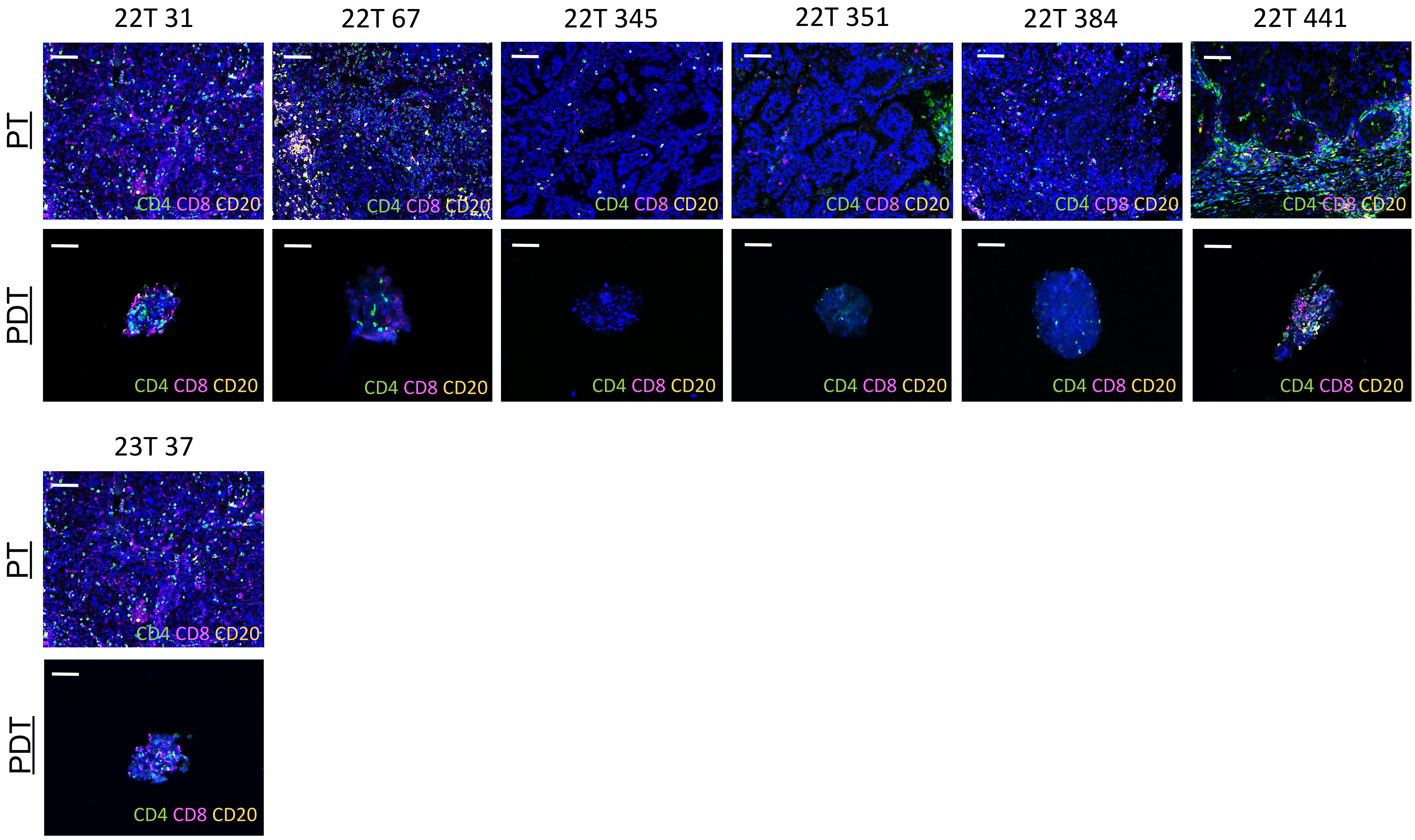

Supplementary figure 7. Related to figure 2. IHC staining of CD4, CD8 and CD20 biomarkers on primary tumoral tissues (PT) and PDTs . Nuclei was stained with DAPI. Scale bar 100µm

VACV **Kegg pathways**

PDTs

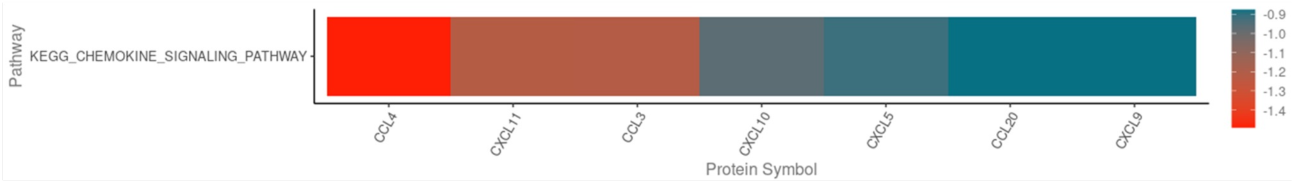

PDHOs

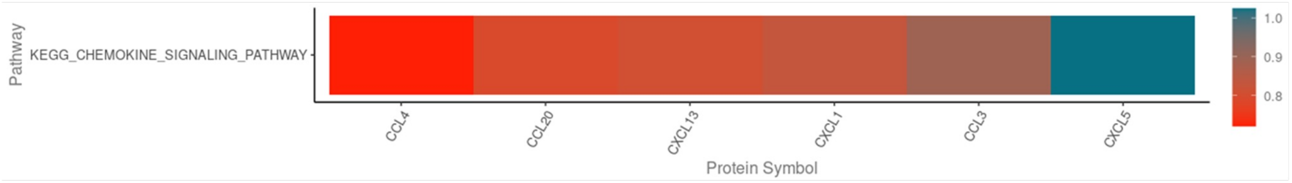

VACV GM-CSF +

PDTs

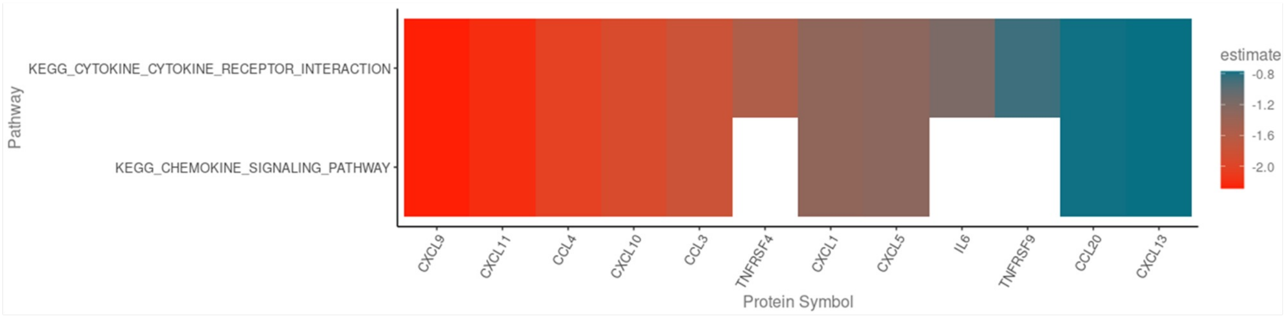

PDHOs

No significantly enriched pathways

Supplementary figure 8. Related to Figure 4. A. Gene set enrichment analysis (GSEA) (KEGG analysis) performed for each comparison VACV or VACV GM-CSF+ versus control, in PDTs and PDHOs n=5. Only the significantly enriched pathway with adjusted p-value ≤ 0.1 are reported on the figure.

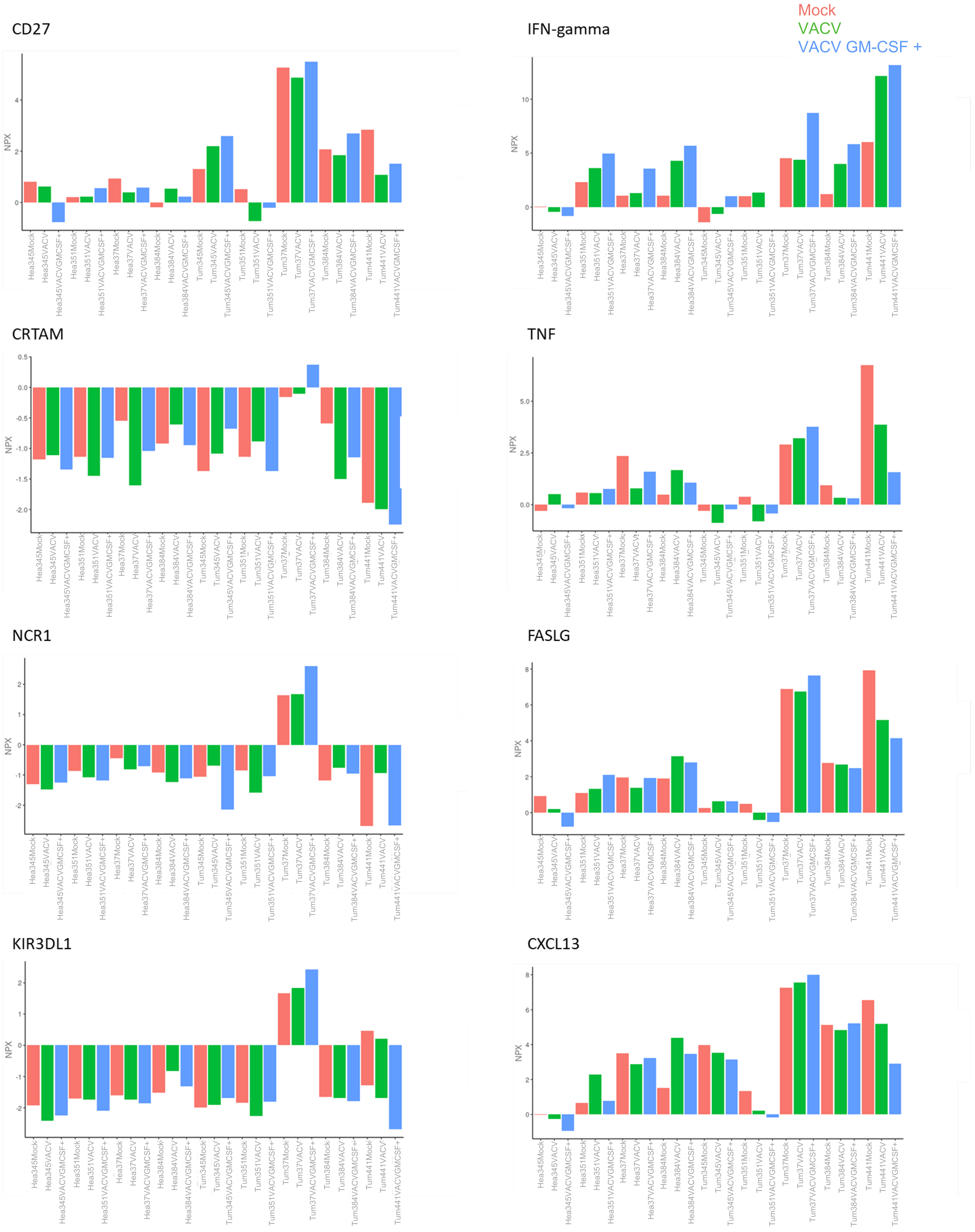

Supplementary figure 8. Related to Figure 4. B. Proteins of interest expression across all patients. These proteins were identified as immunostimulatory ones.

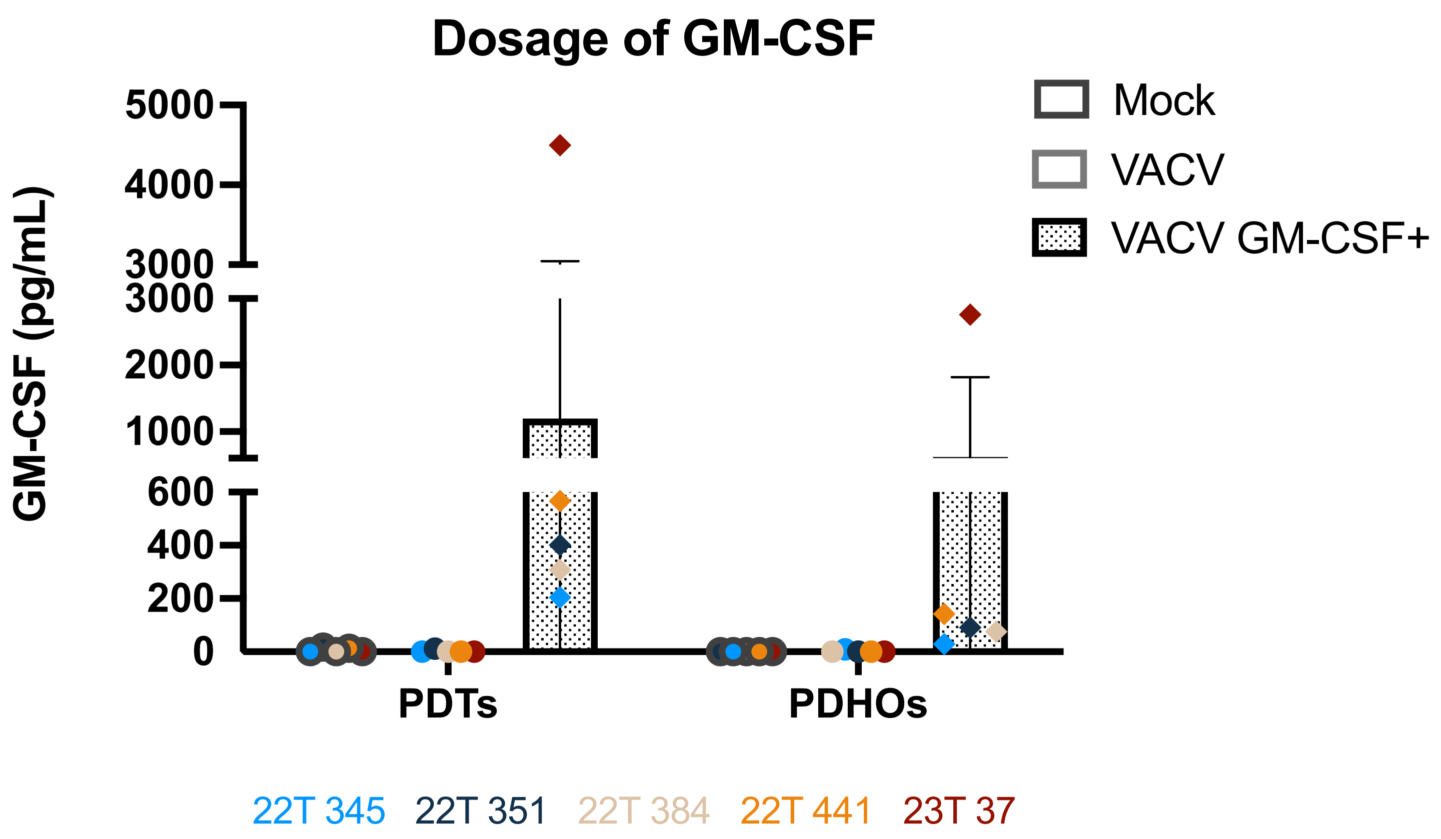

Supplementary Figure 9. Related to Figure 4. GM-CSF expression in PDTs and PDHOs lysates after oncolytic viruses infection were measured by Procarta Plex assay (t-test was performed to test significativity and p>0;05 : ns)

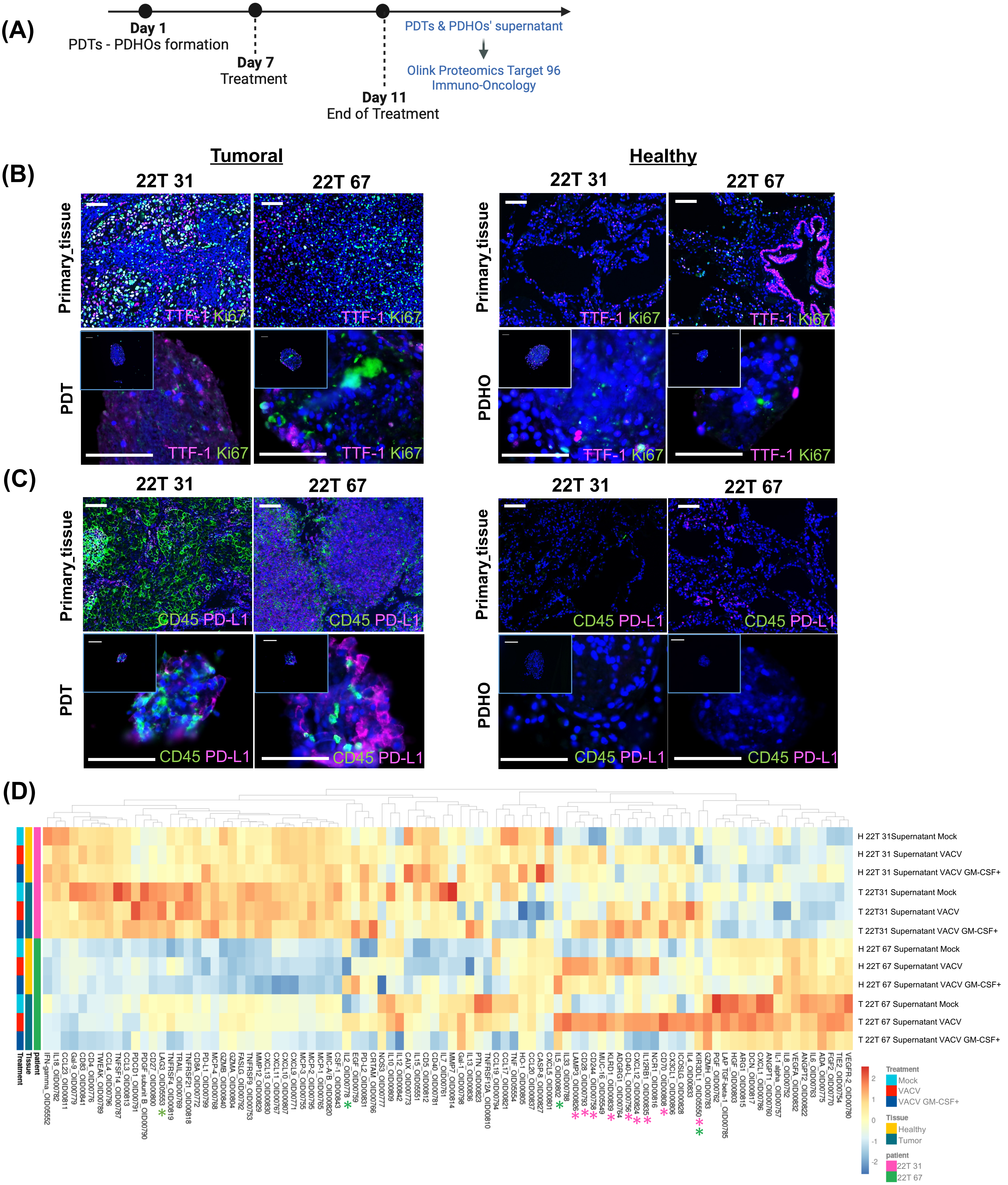

Supplementary Figure 10. Related to Figure 4. Analysis of secreted proteins showed some immunomodulatory effects of VACV GM-CSF+ in PDTs and PDHOs

(A) Workflow of PDTs and PDHO for proteomic' analysis in the supernatant.

(B) IHC of biomarkers TTF-1 / Ki-67 expression. Scale bar 100µm

(C) IHC of biomarkers CD45 / PD-L1 expression. Scale bar 100µm

(D) Heatmap showing the expression of detectable proteins based on a z-score and on a clustering cluster_rows=FALSE. n=2 patients
