# Supplementary material for: Patient-derived tumoroids and proteomic signatures: tools for early drug discovery
Source: Front Immunol. 2024 Apr 18;15:1379613. doi: 10.3389/fimmu.2024.1379613 (PMC11063793; doi:10.3389/fimmu.2024.1379613)
Supplement: Supplementary file 2 [file Table_1.docx]

| Patient identity | Tobacco status | Gender | Age | NSCLC subtype, TNM score and stage | Status PD-L1 |
| --- | --- | --- | --- | --- | --- |
| 22T 31 | Past smoker | Male | 71 | Adenocarcinoma  pT3N2M0  Stage IIIB | 100% |
| 22T 67 | Past smoker | Male | 80 | Adenocarcinoma  pT4N2M0  Stage IIIB | 90% |
| 22T 345 | Current smoker | Male | 65 | Adenocarcinoma  pT3N2M0  Stage IIIB | 0% |
| 22T 351 | Past smoker | Female | 67 | Adenocarcinoma  pT4N0M0  Stage IIIA | Not done |
| 22T 384 | Current smoker | Male | 72 | Adenocarcinoma  pT1cN0M0  Stage IA3 | Not done |
| 22T 441 | Past smoker | Male | 83 | Adenocarcinoma  pT3N0M0  Stage IIB | 60% |
| 23T 37 | Past smoker | Male | 69 | Adenocarcinoma  pT3N0M0  Stage IIB | 90% |

pT = Primary Tumor

pT1c: tumor>2cm but ≤ 3 cm

pT3: tumor > 5 cm but ≤ 7 cm or invasion of parietal pleura (PL3), chest wall, phrenic nerve or parietal pericardium, or presence of a separate tumor nodule in the same lobe

N: Regional lymph node

M: Distant metastasis

**Supplementary table S1. List of lung cancer patients’ samples**
